# Reproductive carrier screening among Chinese couples experiencing unexplained recurrent pregnancy loss

**DOI:** 10.1186/s12884-026-09571-7

**Published:** 2026-06-30

**Authors:** Honglei Duan, Zihan Jiang, Ying Zhang, Chunxiang Zhou, Jie Li

**Affiliations:** 1 Center for Obstetrics and Gynecology, Nanjing Drum Tower Hospital, Affiliated Hospital of Medical School, Nanjing University, Nanjing, China; 2 Center for Obstetrics and Gynecology, Nanjing Drum Tower Hospital Clinical College of Nanjing Medical University, Nanjing, China

**Keywords:** Carrier screening, Recurrent pregnancy loss, DNA Sequencing, Pathogenic variants, Autosomal recessive inheritance

## Abstract

**Background:**

To investigate the prevalence of pathogenic/likely pathogenic (P/LP) variants in 485 genes associated with autosomal recessive and X-linked disorders among Chinese couples experiencing unexplained recurrent pregnancy loss (RPL).

**Methods:**

From March 2021 to December 2024, 48 couples with unexplained RPL and 100 control couples were recruited. A carrier screening panel covering 485 genes associated with 540 autosomal recessive and 84 X - linked hereditary diseases was used. Genomic DNA sequencing was performed, and variants were interpreted based on the American College of Medical Genetics and Genomics (ACMG) classification system.

**Results:**

Among the RPL couples, 93.8% (45/48) carried at least one P/LP variant, and 54.2% (26/48) carried three or more. High carrier rates were observed for *GJB2* (1/12), *DUOX2* (1/14), *SRD5A2* (1/14), *ABCA4* (1/16), and *UGT1A1* (1/16). The carrier frequency of *SRD5A2* variants was significantly higher in RPL females than in controls (*P* < 0.05). Two couples were identified as high-risk for offspring with phenylketonuria (PKU) or spinal muscular atrophy (SMA).

**Conclusions:**

Chinese couples with unexplained RPL exhibit a high prevalence of P/LP variants, particularly in metabolic and developmental genes. These findings indicate potential genetic contributions to RPL pathogenesis and highlight the clinical utility of carrier screening for reproductive counseling. Future research should explore the roles of heterozygous variants in pregnancy loss and validate the associations in larger cohorts.

**Supplementary Information:**

The online version contains supplementary material available at https://doi.org/10.1186/s12884-026-09571-7.

## Background

Recurrent pregnancy loss (RPL), clinically defined as two or more consecutive pregnancy losses before 20 weeks of gestation, affects approximately 1–2% of reproductive-aged couples [1]. The known risk factors for RPL encompass maternal anatomical abnormalities, pre-thrombotic states, immune dysfunctions, uterine structural anomalies, infectious agents, and embryonic genetic aberrations [2]. Cytogenetic studies have demonstrated that over 50% of early spontaneous abortions result from genetic abnormalities, predominantly chromosomal numerical or structural abnormalities in the developing embryo. Genetic analysis of embryonic tissues and maternal risk factors screening have been utilized to explore the potential causes of pregnancy loss. Nevertheless, approximately 30% of RPL cases in clinical practice remain unexplained [3].

Besides chromosomal abnormalities in the embryo, monogenic diseases have long been postulated as genetic contributors to pregnancy loss. Certain genetic disorders are known to be associated with in-utero fetal demise, such as Barth syndrome (OMIM 302060) and lethal multiple pterygium syndrome (OMIM 253290) [4, 5]. However, a well-defined screening protocol for monogenic genetic disorders specifically designed for couples experiencing recurrent pregnancy loss remains to be established.

Reproductive carrier screening aims to identify individuals or couples at risk of having a child affected by an autosomal recessive or X-linked genetic disorder. The American College of Medical Genetics and Genomics (ACMG) and the American College of Obstetricians and Gynecologists (ACOG) have issued position statements to guide the clinical use of reproductive carrier screening [6–8]. A tier - based carrier screening system has been established to ensure equitable access to testing with enhanced precision.

Reproductive-age couples experiencing unexplained RPL are more anxious and are inclined to opt for carrier screening to reduce the risk of having a child with a genetic disorder. However, the reproductive carrier screening used in clinical practice primarily targets the autosomal recessive or X-linked genetic disorders associated with shortened lifespan during infancy or childhood, intellectual disability, death in early adulthood, impaired mobility, or a malformation involving an internal organ [9–11]. Given this focus, the additional value of such screening for couples with RPL remains unclear, as the relationship between these specific genetic disorders and the etiology of RPL has not been firmly established.

This study was designed to conduct a retrospective analysis of Chinese couples experiencing unexplained RPL, aiming to investigate the prevalence of pathogenic (P) or likely pathogenic (LP) variants in 485 genes associated with 540 autosomal recessive hereditary diseases and 84 X-linked hereditary diseases. Through systematic screening of these couples, the study sought to elucidate the potential genetic contributions to RPL and characterize the carrier frequencies of these variants.

## Methods

### Research subjects

From March 2021 to December 2024, 48 couples experiencing two or more spontaneous abortions were recruited at the Obstetrics and Gynecology Center of Nanjing Drum Tower Hospital in Nanjing, China. All couples underwent at least one chromosome karyotype analysis or copy number variation analysis on embryonic tissues, and no genetic abnormalities were identified in either the embryonic tissues or the couples themselves. Information on demographics, adverse birth history, medical history, and family history of the couples were collected. None of the couples were in consanguineous marriages. All RPL couples were strictly defined as having unexplained RPL, with systematic exclusion of those presenting known risk factors (e.g., anatomical abnormalities, pre-thrombotic states, immune dysfunctions, endocrine disorders, infections, or a family history of genetic disorders). The 100 control couples had no adverse birth history, no relevant medical history, and no family history of genetic disorders.

Approximately 3 mL peripheral blood from the couples was collected in EDTA vacutainer tubes (Becton Dickenson). This study was approved by the Ethics Committee of Nanjing Drum Tower Hospital (No.2021-464-02), and written informed consent was obtained from all participating couples.

### Carrier screening panel

The carrier screening panel was constructed according to ACMG suggestions and List of China rare diseases as well as the local population variation database. A total of 485 genes associated with 540 autosomal recessive hereditary diseases and 84 X-linked hereditary diseases were included. The selection of conditions for the carrier screening panel was guided by three key criteria [12] : (1) adherence to current guidelines from the ACMG and other relevant professional societies, restricting to conditions exhibiting a carrier frequency > 1/200 in any population; (2) clinical relevance, encompassing diseases with moderate to severe phenotypes that lead to significant lifelong morbidities or mortalities; and (3) technical feasibility, requiring genes to be detectable by Next Generation Sequencing (NGS) with defined technical details and results verifiable through orthogonal methods (e.g., Sanger sequencing).

The diseases covered by the screening panel encompassed endocrine and metabolic diseases (157/624, 25.2%), nervous system diseases (132/624, 21.2%), multiple system diseases (104/624, 16.7%), ophthalmology and otorhinolaryngology diseases (68/624, 10.9%), blood and immune system diseases (45/624, 7.2%), lysosomal disorder (32/624, 5.1%), musculoskeletal and connective tissue diseases (21/624, 3.4%), skin disorders (20/624, 3.2%), and other diseases (45/624, 7.2%). The analysis of genes associated with X-linked hereditary diseases was performed exclusively in female participants. The list of genes and associated diseases is presented in Additional file 1.

### Genomic DNA sequencing and variant interpretation

Genomic DNA was extracted from peripheral blood using a DNA extraction kit (Qiagen, GmbH, Germany). Captured libraries (KAPA, Roche, Swiss) were sequenced with 2 × 150 bp reads on the NextSeq550Dx platform (Illumina, San Diego, CA, USA). Quality control and trimming of raw data were performed using FastQC (v0.11.5) software. Sequence reads were mapped onto the human reference genome hg37 using the BWA tool. The single nucleotide variants, insertions, deletions, and copy number variations covered more than two continue exons were called and annotated by ISO genetics software (Fujun Genetics Biotechnology, China). The average coverage depth was > 100×, with over 99% of the target regions covered by at least 20 reads. Evaluate pathogenic gene variation based on the ACMG classification system [13]. Only pathogenic (P) and likely pathogenic (LP) variants were reported as positive results.

### Statistical analysis

Statistical analysis was performed using SPSS version 26.0 (IBM Corp., Armonk, NY, USA). Continuous variables were expressed as mean ± SD or median (interquartile range) based on normal distribution assessed by the Shapiro-Wilk test. Categorical variables were presented as numbers (proportions), and intergroup comparisons were conducted using the chi-square test or Fisher’s exact test, as appropriate. A two-sided *P* < 0.05 was considered statistically significant.

## Results

**Fig. 1:**
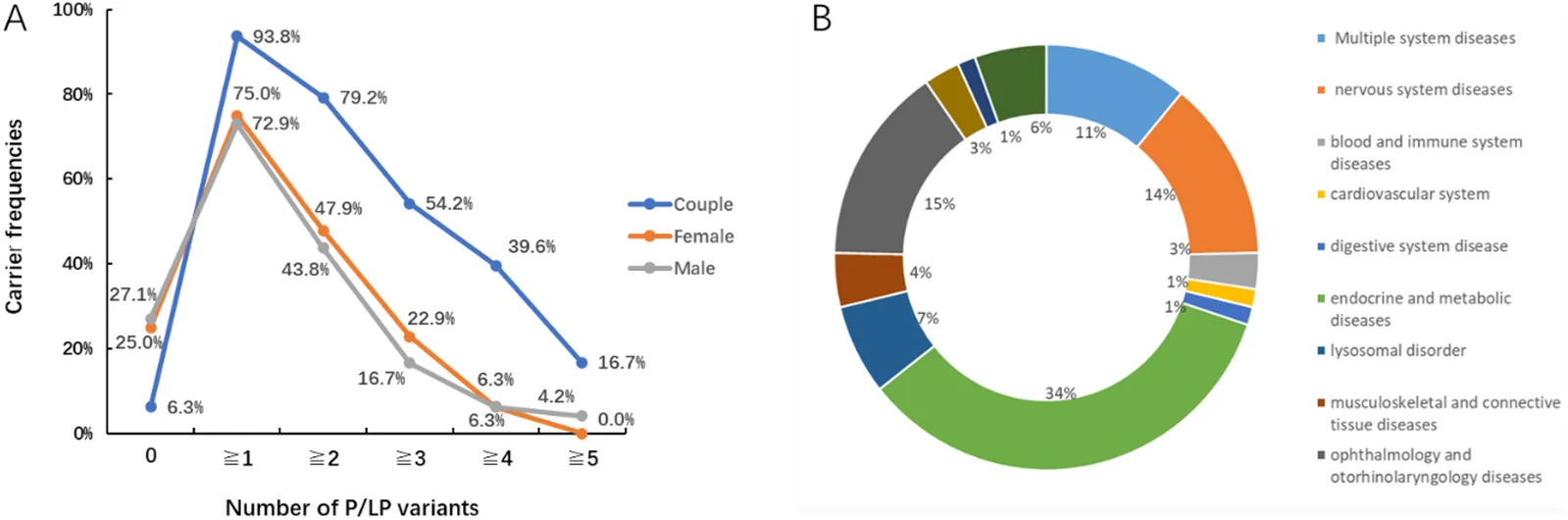
Carrier frequencies and Disease spectrum. **A** Carrier rate of pathogenic/likely pathogenic variants in couples with recurrent pregnancy loss; **B** Disease spectrum associated with pathogenic/likely pathogenic variants in couples with recurrent pregnancy loss

Among 48 couples with RPL, the mean age of females was 29.65 ± 2.76 years, the mean age of males was 30.45 ± 3.05 years. The number of spontaneous abortions before 20 weeks of gestation ranged from 2 to 7. Specifically, 29 couples experienced 2 miscarriages, 14 couples experienced 3, 3 couples experienced 4, 1 couple experienced 5, and 1 couple experienced 7, for a total of 124 miscarriages. The mean number of miscarriages per couple was 2.58 ± 0.96. Detailed demographic and clinical characteristics of both RPL and control couples are presented in Table 1. A total of 143 P/LP variants were identified in 45 couples, indicating that approximately 93.8% (45/48) of the couples carried at least one P/LP variant. Furthermore, more than half of the couples (54.2%, 26/48) carried three or more P/LP variants (Fig. 1A). These 143 variants were distributed across 73 genes, with 34% (25/73) of the genes associated with endocrine and metabolic diseases, 15% (11/73) linked to ophthalmology and otorhinolaryngology diseases, and 14% (10/73) related to nervous system disorders (Fig. 1B). A complete list of all 73 genes harboring P/LP variants detected in the RPL group, along with their associated diseases, inheritance patterns, number of cases identified, is provided in Additional file 2.

**Table 1:** Baseline characteristics of RPL couples (*n* = 48) and control couples (*n* = 100)

Characteristic	RPL Couples (*n* = 48)	Control Couples (*n* = 100)	*P* value
Female age (years), mean ± SD	29.65 ± 2.76	30.67 ± 4.00	0.119
Male age (years), mean ± SD	30.45 ± 3.05	31.60 ± 3.34	0.071
Number of spontaneous abortions, mean ± SD	2.58 ± 0.96	0	NA
Gestational age at loss (weeks), mean ± SD	7.14 ± 1.69	NA	NA

*NA* Not applicable

Among couples experiencing unexplained recurrent pregnancy loss, the genes with the highest carrier rates were *GJB2* (1 in 12), *DUOX2* (1 in 14), *SRD5A2* (1 in 14), *ABCA4* (1 in 16), and *UGT1A1* (1 in 16) (Table 2). Eight P/LP variants in *GJB2* were detected in seven patients, with the c.109G > A (p.Val37Ile) variant representing the most frequent mutation, detected in six cases. One male without clinical manifestations of hearing impairment was determined to be a compound heterozygote for *GJB2* pathogenic variants: c.109G > A (p.Val37Ile) and c.235del (p.Leu79Cysfs*3). According to the ACMG guidelines, the c.109G > A variant is classified as pathogenic; however, it has also been reported to exhibit reduced penetrance and is associated with a late-onset or mild clinical phenotype [14, 15]. The male individual, identified as a compound heterozygote for c.109G > A and c.235del, currently shows no detectable hearing abnormalities, likely attributable to the variable penetrance of the c.109G > A variant.

**Table 2:** The first five pathogenic/likely pathogenic genes were detected in RPL group

NO.	Gene	Disease	Inheritance	Number of cases identified in RPL group		Carrier frequency		
				Female	Male	RPL group	control group	*P* value
1	*GJB2*	Nonsyndromic hearing loss, GJB2-related	AR	2	6	1/12	1/9	0.303
2	*DUOX2*	Thyroid dyshormonogenesis 6	AR	4	3	1/14	1/12	0.127
3	*SRD5A2*	Pseudovaginal perineoscrotal hypospadias	AR	6	1	1/14	1/67	< 0.05
4	*ABCA4*	Stargardt disease 1; Cone-rod dystrophy 3	AR	3	3	1/16	1/67	0.062
5	*UGT1A1*	Crigler-Najjar syndrome	AR	4	2	1/16	1/12	0.561

Statistical analysis revealed no significant differences in carrier frequencies of P/LP variants in *GJB2*, *DUOX2*,* ABCA4*, and *UGT1A1* between RPL couples and the control cohort (*n* = 100), except for *SRD5A2* (*P* < 0.05). In total, seven *SRD5A2* variants were detected in six females and one male. These variants encompassed four c.680G > A (p.Arg227Gln) variants, two c.196G > A (p.Gly66Arg) variants, and one c.433 C > T (p.Arg145Trp) variant. The carrier frequency of *SRD5A2* variants among females with RPL was markedly higher than that in the control group (*P* = 0.001).

Two couples were identified as carriers at an elevated risk of having an affected baby with autosomal recessive genetic disorders. One couple, with a history of two pregnancy losses, were both carriers of a *PAH* pathogenic variant. The fetal tissues from their second abortion were proven to be a compound heterozygote of *PAH* pathogenic variants, NM_000277.3:c.611 A > G (maternal) and NM_000277.3:c.913–7 A > G (paternal). Another couple were carriers of the *SMN1* pathogenic variant (NM_000344.3:ex.7-8del). The abortion tissue of this couple was identified as having a normal chromosome karyotype, yet the carrier status for *SMN1* variants remained unclear.

## Discussion

Chromosomal aneuploidy represents the most prevalent genetic cause of spontaneous miscarriage. Nevertheless, within cases of recurrent miscarriage, the incidence of chromosomal aneuploidy diminishes as the number of miscarriages rises [16]. This decline suggests that other underlying etiologies likely contribute to recurrent pregnancy loss in affected couples. A systematic review of 428 case-control studies identified an association between unexplained recurrent miscarriage and 21 variants in parental genes involved in immune response, coagulation, metabolism, and angiogenesis [17]. Despite the evidence remains predominantly low-certainty, it underscores potential genetic contributors to the etiology of recurrent pregnancy loss and provides a foundation for future research directions and the development of targeted diagnostic and therapeutic interventions. In this study, a 485-gene panel was utilized to conduct carrier screening on 48 couples with recurrent pregnancy loss of undetermined etiology. The 485-gene panel is linked to 540 autosomal recessive hereditary diseases and 84 X-linked hereditary diseases, including severe, highly prevalent conditions affecting endocrine, metabolic, hematologic, and immune systems. Our analysis identified 143 distinct variants among the study cohort, with a mean carrier frequency of 1.5 variants per individual. More than half of the couples (54.2%) were found to carry three or more P/LP variants.

In this study, *GJB2*, *DUOX2*, *SRD5A2*,* ABCA4*, and *UGT1A1* were found to exhibit relatively high carrier rates among couples experiencing recurrent pregnancy loss. A significantly higher carrier frequency of *SRD5A2* variants was observed in couples experiencing recurrent pregnancy loss compared to the normal controls, particularly in female individuals. The *SRD5A2* gene encodes the 5α-reductase type 2 (5α-RD2) enzyme, a key enzyme in steroid metabolism that catalyzes the conversion of testosterone into dihydrotestosterone [18, 19]. In 46,XY individuals, *SRD5A2* variants resulting in the absence or defects of the 5α-RD2 isoenzyme cause incomplete intrauterine masculinization of the external genitalia [20, 21]. In contrast, 46,XX individuals carrying mutations in the *SRD5A2* gene are scarcely reported. Three documented cases of 46,XX females homozygous for the p.Arg246Trp exhibited delayed menarche and low androgen levels, yet all remained fertile [22]. The c.680G > A (p.Arg227Gln) and c.433 C > T (p.Arg145Trp) variants, identified in couples with recurrent pregnancy loss, have been shown to severely reduce enzyme activity to 3.2% and 6% of wild-type levels, respectively [18]. The *SRD5A2* rs523349 (Val89Leu) polymorphism has been reported to be significantly associated with miscarriage risk [23]. However, the precise role of testosterone/dihydrotestosterone imbalance in female reproductive physiology remains incompletely understood, highlighting the need for further research to better elucidate its implications for fertility and pregnancy maintenance.

In addition, two couples were identified as being at a 1/4 risk of conceiving offspring affected by spinal muscular atrophy (SMA) or phenylketonuria (PKU), as both partners in each couple were carriers of P/LP variants in the *SMN1* or *PAH* genes, respectively. One aborted fetus from the couple carrying *PAH* variants was confirmed to be a compound heterozygote for *PAH* pathogenic variants, indicating that the fetus would have been affected by PKU. For the couple with *SMN1* pathogenic variants, chromosomal karyotype analysis of a miscarried embryo revealed no abnormalities; however, *SMN1* gene variant testing was not carried out. Both PKU and SMA have been frequently reported in live births, suggesting that these conditions are not universally lethal during intrauterine development [24, 25]. Given the limited understanding of the impact of these pathogenic variants on early embryonic development, it cannot be conclusively determined whether the compound heterozygous variants in the *PAH* gene were directly responsible for the spontaneous miscarriage. Nevertheless, the identification of carrier status in these families is of paramount importance, as it provides critical information for guiding future reproductive planning.

According to Mendel’s Law of Dominance, carriers of pathogenic variants associated with recessive genetic disorders typically do not exhibit clinical phenotypes due to the presence of a normal allele that compensates for the defective one. However, emerging evidence suggests that heterozygous genotypes for certain recessive diseases can manifest intermediate phenotypic expression. This phenomenon of intermediate phenotypes has been documented in carriers of conditions such as PKU, homocystinuria, galactosemia, and gyrate atrophy of the choroid and retina, highlighting the potential for certain clinical manifestations even in the absence of full disease expression [26, 27]. For instance, liver biopsy studies have revealed that *PAH* P/LP variant carriers exhibit only 7.3% to 10% of phenylalanine hydroxylase enzymatic activity compared to individuals without P/LP variants [28]. During pregnancy, elevated maternal phenylalanine levels can significantly impair fetal health, as phenylalanine is actively transported across the placenta. In the offspring of mothers with untreated PKU, 92% experience developmental delay or intellectual disability, 73% present with microcephaly, and 12% are affected by congenital heart defects [29]. Such pregnancies are also associated with an increased risk of spontaneous abortion, postnatal growth restriction, and craniofacial abnormalities. Emerging evidence suggests a potential correlation between *PAH* carrier status and reduced phenylalanine metabolism, as well as associations with mental health issues and an elevated risk of melanoma [30, 31]. However, the relationship between *PAH* carrier status and pregnancy loss remains underexplored and warrants further investigation to clarify potential causal links.

The relatively small sample size in this single-center study limits the generalizability of the findings. Additionally, while the study cohort consisted of Han Chinese couples without consanguineous relationships, carrier frequencies and variant spectra may vary across different endogamous populations. Future research should validate these associations in larger cohorts and diverse ethnic populations, as well as explore the mechanistic roles of heterozygous variants in pregnancy loss, to refine screening protocols and improve outcomes for affected couples.

## Conclusions

In summary, this study highlights the potential genetic contributions to unexplained recurrent pregnancy loss among Chinese couples, revealing a high prevalence (93.8%) of P/LP variants in a 485-gene carrier screening panel. Two couples were identified as high-risk carriers for autosomal recessive disorders (PKU and SMA), underscoring the clinical utility of screening for reproductive counseling. Notably, *SRD5A2* variants are significantly more frequent in RPL females than controls. Future research should explore the mechanistic roles of heterozygous variants in pregnancy loss and validate these associations in larger cohorts to refine screening protocols and improve outcomes for affected couples.

## Supplementary Information

Supplementary Material 1.

Supplementary Material 2.

## Data Availability

The data that support the findings of this study are available on request from the corresponding author.

## Abbreviations

ACMG: American College of Medical Genetics and Genomics
ACOG: American College of Obstetricians and Gynecologists
LP: Likely pathogenic
P: Pathogenic
PKU: Phenylketonuria
SMA: Spinal muscular atrophy
NGS: Next Generation Sequencing
RPL: Recurrent pregnancy loss
